# SEanalysis: a web tool for super-enhancer associated regulatory analysis

**DOI:** 10.1093/nar/gkz302

**Published:** 2019-04-27

**Authors:** Feng-Cui Qian, Xue-Cang Li, Jin-Cheng Guo, Jian-Mei Zhao, Yan-Yu Li, Zhi-Dong Tang, Li-Wei Zhou, Jian Zhang, Xue-Feng Bai, Yong Jiang, Qi Pan, Qiu-Yu Wang, En-Min Li, Chun-Quan Li, Li-Yan Xu, De-Chen Lin

**Affiliations:** 1 School of Medical Informatics, Daqing Campus, Harbin Medical University, Daqing 163319, China; 2 Institute of Oncologic Pathology, Medical College of Shantou University, Shantou 515041, China; 3 Guangdong Province Key Laboratory of Malignant Tumor Epigenetics and Gene Regulation, Sun Yat-Sen Memorial Hospital, Sun Yat-Sen University, Guangzhou 510120, China

## Abstract

Super-enhancers (SEs) have prominent roles in biological and pathological processes through their unique transcriptional regulatory capability. To date, several SE databases have been developed by us and others. However, these existing databases do not provide downstream or upstream regulatory analyses of SEs. Pathways, transcription factors (TFs), SEs, and SE-associated genes form complex regulatory networks. Therefore, we designed a novel web server, SEanalysis, which provides comprehensive SE-associated regulatory network analyses. SEanalysis characterizes SE-associated genes, TFs binding to target SEs, and their upstream pathways. The current version of SEanalysis contains more than 330 000 SEs from more than 540 types of cells/tissues, 5042 TF ChIP-seq data generated from these cells/tissues, DNA-binding sequence motifs for ∼700 human TFs and 2880 pathways from 10 databases. SEanalysis supports searching by either SEs, samples, TFs, pathways or genes. The complex regulatory networks formed by these factors can be interactively visualized. In addition, we developed a customizable genome browser containing >6000 customizable tracks for visualization. The server is freely available at http://licpathway.net/SEanalysis.

## INTRODUCTION

Super-enhancers (SEs), composed of clusters of enhancers, regulate cell-type-specific expression programs through a unique transcriptional activity to drive expression of genes that define cell identity (1–3). Because of their prominent functions in transcriptional regulation, SEs have been annotated in numerous cell/tissue types. As a hallmark of cancer, the alterations of signaling pathways converge on regulating terminal DNA-bound transcription factors (TFs) (4,5). Importantly, SEs are more frequently occupied by terminal TFs of pathways than typical enhancers. Concordantly, SE-associated genes are also more responsive to signalling cues than typical enhancers (5). Pathways, TFs, SEs and SE-associated genes form complex regulatory networks (5–7). These regulatory networks allow SEs to act as a crucial platform for pathways to regulate gene expression programs with much higher potency than typical enhancers. Notably, the functional interplay between oncogenic pathways and SEs is particularly prominent in regulating cancer biology, which have been highlighted by numerous reports (5,8–10).

**Figure 1.**
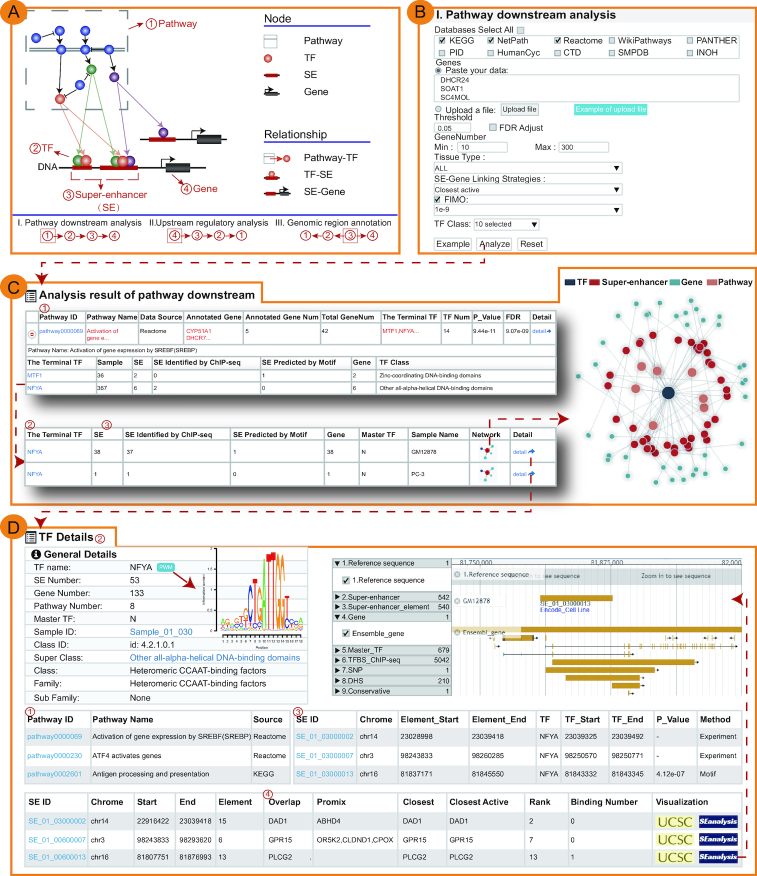
Main functions of SEanalysis. (**A**) Schematic diagram of SEanalysis core functions. (**B**) Input and parameter page of ‘Pathway downstream analysis’. (**C**) Results page of ‘Pathway downstream analysis’. (**D**) Detailed interactive table of results of ‘Pathway downstream analysis’.

Several SE databases have been developed, including dbSUPER (11), SEA (12) and SEdb (13). These databases summarize and catalog SE regions for various tissue and cell types using an H3K27ac signal-based ranking method (ROSE) (14). However, none of the databases provide downstream or upstream regulatory analysis involving SEs. To address this need, we developed the SEanalysis web server to provide SE-associated regulatory analyses. Users can perform several SE-associated analyses in our web server. I. Pathway downstream analysis: with the input of a set of genes of interest, SEanalysis will identify pathways that they are significantly enriched in, the terminal TFs that are downstream of the identified pathways, the SEs and SE-associated genes occupied by the terminal TFs (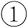
→
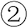
→
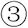
→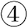 in Figure 1A). II. Upstream regulatory analysis: with the input of gene(s) of interest, SEanalysis will identify associated SEs and determine which TFs occupy these SE regions and the upstream pathways of the identified TFs (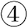
→
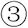
→
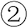
→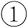 in Figure 1A). III. Genomic region annotation: users can input genomic region(s) of interest in bed format to discover SEs overlapping the region(s), SE-associated genes and TFs occupying the SE regions and the upstream pathways of identified TFs (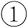
←
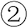
←
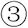
→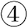 in Figure 1A).

## DESCRIPTION OF WEB SERVER

### Annotation of SEs and SE-associated genes

We obtained more than 330 000 SE regions involving 542 cells/tissues from the SEdb database (13) that was developed by our group using H3K27ac ChIP-seq data from NCBI GEO/SRA (15), ENCODE (16), Roadmap (16,17) and GGR (Genomics of Gene Regulation Project) (16). Raw sequencing reads were aligned to hg19 reference genomes with Bowtie (v0.12.9) (1,18), peaks were called using MACS14 (v1.4.2) (19), and SE regions were annotated using ROSE (14) software. Four different strategies were used to annotate SE-associated genes: closest active genes (20), overlapping genes, proximal genes and the closest genes (14).

### Identification of TF occupancy in SE regions

**Figure 2.**
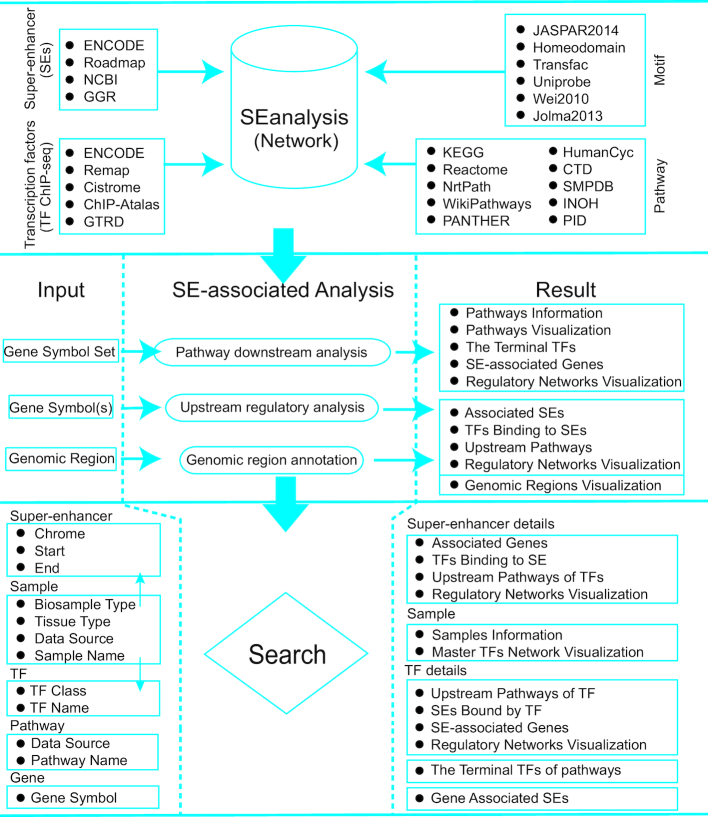
SEanalysis content and construction. SEanalysis contains a large number of SEs, TF ChIP-seq data, and DNA-binding sequence motifs as well as pathway information. Users can perform the following SE-associated analyses in our web server: I. Pathway downstream analysis, II. Upstream regulatory analysis, and III. Genomic region annotation. SEanalysis supports five searching modes, including ‘Searching by SE’, ‘Searching by Sample’, ‘Searching by TF’, ‘Searching by Pathway’ and ‘Searching by Gene’.

To identify TFs binding to SEs, we collected a total of 5042 TF ChIP-seq datasets from ENCODE (16), Remap (21), Cistrome (22), ChIP-Atlas (http://chip-atlas.org) and GTRD (23) (Figure 2, top panel). For the uniformity of format and version, these peak datasets were converted to the hg19 genome using liftOver (http://genome.ucsc.edu/cgi-bin/hgLiftOver) tool of UCSC (24), and peaks that were failed to be converted were discarded. We used the ‘cat’ shell command to merge files of different samples for the same TF from the same tissue to generate union sets of peaks. TF binding peaks overlapping with constituent enhancers of SEs in matched cell/tissue types were identified using BEDTools (v2.25.0) (25). Motif occurrences in constituent enhancers of SEs for ∼700 TFs were identified using FIMO (Find Individual Motif Occurrences) (26) from the MEME (Multiple Em for Motif Elicitation) suite (27). More than 3000 DNA binding motifs for ∼700 TFs were compiled from the TRANSFAC (28) and MEME suite (20,27), based on the following collections: JASPAR CORE 2014 vertebrates (29), Jolma2013 (30), Homeodomains (31), UniPROBE (32), Wei2010 (33). Finally, TF motif occurrence within SE constituents was identified with a *P*-value threshold of 1e–5.

### Identification of master TFs and classification of TFs

Saint-André *et al.* developed CRC_Mapper program to efficiently reconstruct cell-type-specific core regulatory circuitry (CRC) models based on the identification of SE-associated master TFs in a number of cell types (20). In this program, master TFs are defined as auto-regulated TFs encoded by SE-associated genes (1,2,20) that bind to at least three DNA sequence motifs at SEs associated with their own gene, and form fully interconnected auto-regulatory loops with other auto-regulated TFs by binding to SEs associated with other TFs within the loop (34–37). We identified master TFs for each cell/tissue using this program and provided interactive visualization of the CRC model. In addition, we manually assigned four generic level classifications (superclass, class, family and subfamily) of TFs according to TFClass database (38), based on their DNA-binding domains.

### Construction of SE-associated regulatory networks

The data above were combined to construct an SE-associated regulatory network (Figure 2, top panel). Nodes of this network were composed of pathways, TFs, SEs and SE-associated genes. First, we established relationships between SEs and occupying TFs by either direct evidence generated from TF ChIP-seq data or by prediction based on motif analysis. Next, we obtained 2880 pathways with their pathway components from 10 pathway databases: KEGG, Reactome, NetPath, WikiPathways, PANTHER, PID, HumanCyc, CTD, SMPDB and INOH (39,40). We built relationships between a TF and a pathway if the TF was a component of the pathway. Finally, we constructed SE-associated regulatory networks by merging all relationships between all nodes, including (i) SEs-TFs, (ii) pathways-TFs and (iii) SEs-genes.

### SEanalysis core functions

**Figure 3.**
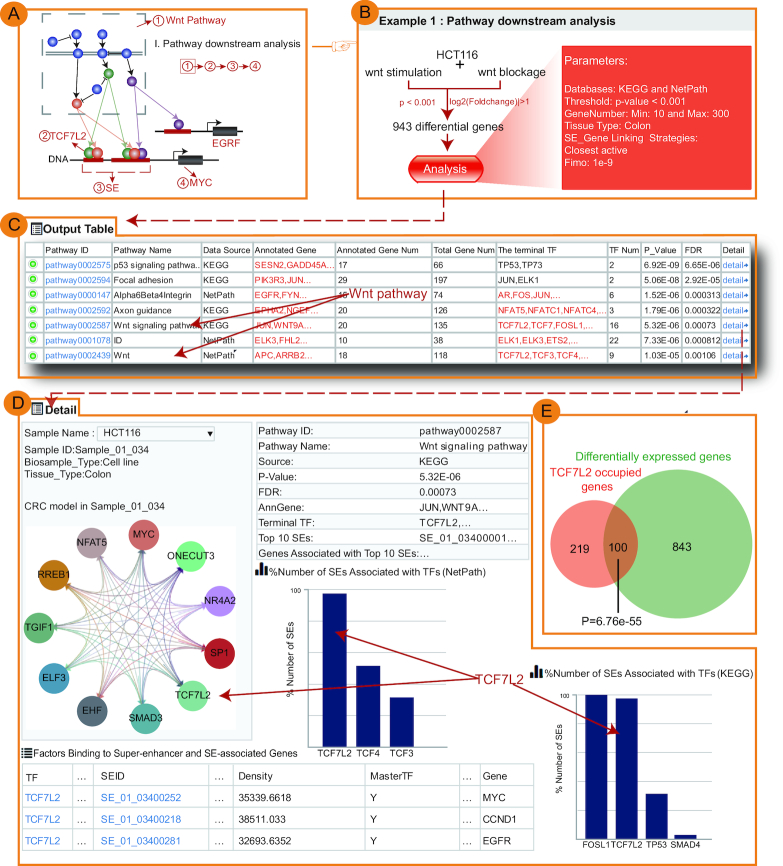
Validation results for ‘Pathway downstream analysis’. (**A**) Schematic diagram of ‘Pathway downstream analysis’. (**B**) Input exemplary data and parameters of ‘Pathway downstream analysis’ using HCT116/Wnt pathway example. (**C**) The output table of ‘Pathway downstream analysis’ generated by our webserver. (**D**) The detailed page of the output table. The page provided the detailed description of the regulatory relationship between TFs involved in the predicted pathway, SEs bound by these TFs, genes associated with SEs, as well as the CRC model. (**E**) SE-associated genes occupied by TCF7L2 were significantly enriched in those exhibiting expression changes upon disruption of Wnt pathway.

We designed three types of analyses to determine SE-associated regulatory networks (Figure 2, middle panel):

*I. Pathway downstream analysis* (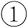
→
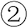
→
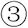
→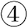 in Figure 1A). With the input of a set of genes of interest and the selection of at least one pathway database (e.g. KEGG), SEanalysis will identify significantly enriched pathways, downstream TFs, SEs occupied by TFs and SE-associated genes (Figure 1B–D). SEanalysis will begin with the identification of the pathways in which these genes are significantly enriched using hypergeometric test (41). For each pathway assuming the entire genome has a total of *n* genes, of which *k* are components of the pathway under investigation, and the set of genes of interest has a total of *s* genes, of which *i* are involved in the same pathway, the enrichment significance *P*-value for that pathway is calculated as:
  \begin{equation*}p\ = {\rm{\ }}1 - \mathop \sum \limits_{x\ = \ 0}^{i - 1} \frac{{\left( {\begin{array}{@{}*{1}{c}@{}} k\\ x \end{array}} \right)\left( {\begin{array}{@{}*{1}{c}@{}} {n - k}\\ {s - x} \end{array}} \right)}}{{\left( {\begin{array}{@{}*{1}{c}@{}} n\\ s \end{array}} \right)}}\end{equation*}
  The false discovery rate (FDR) method is used to correct for multiple testing. Users can adjust the number of genes required to be enriched and set thresholds of *P*-values or FDRs to control the stringency of analysis. SEanalysis offers a ‘FIMO’ option to allow users to set different statistical thresholds to control for false positivity. The ‘SE-Gene Linking Strategies’ option allows users to select different annotation strategies to link SEs with target genes. In addition, ‘Tissue Type’ option allows user to perform targeted analysis in tissues of interest.
  The output table contains basic information of identified pathways (Pathway ID, Pathway name, Pathway source, Annotated gene, Annotated gene number, Total gene number, The terminal TF and TF number), *P*-value and FDR of the enrichment score (Figure 1C). Pathways can be further displayed by clicking the ‘Pathway ID’ button. The TF related statistics will be further viewed by clicking the ‘+’ button, including the number of SEs bound by TF (based on either ChIP-seq data or predicted by motif analysis), the number of genes associated with these SEs and visualization of regulatory networks based on the TF (Figure 1C and D). Furthermore, the ‘detail’ page provides the detailed description of the regulatory relationship between TFs involved in the current pathway, SEs bound by these TFs, and genes associated with SEs (Figure 3).
*II. Upstream regulatory analysis* (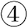
→
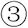
→
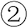
→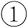 in Figure 1A). With the input of gene(s) of interest and the setting of ‘Tissue Type’, ‘SE-Gene Linking Strategies’, ‘FIMO’ and ‘Pathway Enrichment Threshold’ options, SEanalysis will first identify the associated SEs, then determine the TFs occupying the SE regions and the enriched upstream pathways of the TFs. The output table will show: (i) the relationships between input genes and identified SEs, (ii) the number and names of TFs binding to the SEs (based on either ChIP-seq data or predicted by motif analysis), (iii) master TFs binding to these SEs (predicted by CRC_Mapper) (20) and (iv) upstream pathways and the sample information. The regulatory network base on SEs can be interactively visualized. The ‘detail’ page provides the full description of the regulatory relationship.
*III. Genomic region annotation* (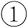
←
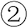
←
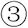
→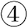 in Figure 1A). Users can upload either a ‘bed’ format file or a list of genomic regions to identify SEs overlapping with the queried regions using Bedtools (25). Furthermore, users can set multiple options, including ‘Tissue Type’, ‘SE-Gene Linking Strategies’, ‘FIMO’ and ‘Pathway Enrichment Threshold’. The output table includes: (i) the identified SEs overlapping with the queried regions and SE-associated genes, (ii) the number and names of TFs binding to the identified SEs (based on either ChIP-seq data or predicted by motif analysis), (iii) the number and names of master TFs binding to the identified SEs and (iv) the number and names of upstream pathways and sample information. The detailed description of the regulatory relationship is provided in the ‘detail’ page.

### Case studies

We used the experimental data from two different studies to validate the key predictions of SEanalysis. For ‘Pathway downstream analysis’ (Figure 3A), we re-analyzed the work wherein a colon cancer cell line (HCT116, known to be dependent on Wnt activation for proliferation) was treated with Wnt inhibitor or stimulator followed by RNA-seq (5). We first obtained 943 differentially expressed genes upon treatment with Wnt modulators from Array Express experiment E-MTAB-651 (*P*-value < 0.001, |log_2_(Foldchange)| > 1, Figure 3B) (5,42). These genes were used as input for our webserver for ‘Pathway downstream analysis’ (parameters: Databases: KEGG and NetPath, Threshold: *P*-value < 0.001, GeneNumber: Min: 10 and Max: 300, Tissue Type: Colon, SE-Gene Linking Strategies: Closest active and FIMO: 1e–9). The output table showed that Wnt pathway was not only significantly enriched (Hypergeometric test; *P*-value = 5.32e–06) but it was also the sole pathway identified by both pathway sources (KEGG and NetPath), and furthermore, it ranked highly as fifth and seventh among all pathways identified (Figure 3C). The webserver next identified a number of terminal TFs downstream of Wnt signaling pathway, including TCF7L2, TCF3, TCF4 and FOSL1. Importantly, using TCF7L2 ChIP-seq generated in HCT116 cells, our analysis showed that TCF7L2 occupied the vast majority of HCT116 SEs (98% of total SEs) (histogram in Figure 3D), which is consistent with the result of Hnisz *et al.* (5) and validated the prediction of webserver. Compared to other terminal TFs of Wnt pathway, TCF7L2 occupied a greater percentage of SEs in both KEGG and NetPath (histogram in Figure 3D). Lastly, we tested whether these SE-associated genes occupied by TCF7L2 were responsive to the manipulation of the Wnt pathway. Notably, these SE-associated genes occupied by TCF7L2 were significantly enriched in those exhibiting expression changes after disruption of Wnt pathway (Hypergeometric test; *P*-value = 6.76e–55) (Figure 3E), again confirming the previous report (5). Some of these TCF7L2-occupied, SE-associated genes included well-established Wnt targets, such as MYC, CCND1 and EGFR. Considering the well-established role of TCF7L2 in mediating Wnt signaling pathway through occupying super-enhancers, these results suggest the value and usefulness of our webserver in linking pathways, terminal TFs and super-enhancer activity.

To validate the prediction of ‘Upstream regulatory analysis’ (Supplementary Figure S1A), we studied luminal breast cancer which is known to be highly and uniquely dependent on estrogen signaling (5). Specifically, we used an ER-positive cell line, MCF-7, wherein a super-enhancer of ESR1 gene has been shown to be occupied by the TF estrogen receptor alpha (ERα). With input of the ESR1 gene in the ‘Upstream regulatory analysis’ (parameters: Tissue Type: Mammary Gland, SE-Gene Linking Strategies: Closest active, FIMO: 1e–9 and Pathway Enrichment Threshold: FDR corrected *P*-value < 0.001) (Supplementary Figure S1B), the output table predicted that the SEs associated with ESR1 gene were indeed occupied by estrogen receptor ERα in almost all ER-positive breast cancer cell lines (Supplementary Figure S1C). Moreover, ERα was further identified as a master TF in multiple ER-positive breast cancer cell lines, along with other well-established ERα interacting TFs, such as XBP1, FOXA1 and GATA3 (Supplementary Figure S1C and D). In the next step of prediction of enriched pathways, the webserver identified that the TFs associated with this ESR1 SE were significantly enriched in pathways including ‘Nuclear receptor transcription pathway (ranked second of all pathways, Hypergeometric test; FDR corrected *P*-value = 5.7e–11)’ and ‘Validated nuclear estrogen receptor alpha network (ranked sixth of all pathways, Hypergeometric test; FDR corrected *P*-value = 5.62e–07)’ (Supplementary Figure S1D, bottom panel). These predictions are congruent with the key role of ERα in mediating nuclear estrogen receptor signaling to the regulation of super-enhancer activity in luminal breast cancer.

Taken together, these data validated all of the key webserver predictions including: (i) pathway enrichment; (ii) terminal TF ranking; (iii) identification of downstream SEs and (iv) annotation of SE-associated genes.

### User-friendly searching and browsing functions

SEanalysis supports five different searching modes: ‘Searching by SE’, ‘Searching by Sample’, ‘Searching by TF’, ‘Searching by Pathway’ and ‘Searching by Gene’. SEanalysis provides data browsing, which is an interactive table with a sorting function that allows users to quickly search for samples and customize filters including ‘Data Sources’, ‘Biosample Type’, ‘Tissue Type’ and ‘Biosample Name’. To further view the SE of a given sample, users can click ‘Sample ID’ button.

### Visualization of regulatory network and customizable genome browser

As mentioned above, SEs, SE-associated genes, TFs binding to SEs and upstream pathways of TFs form complex networks. To facilitate the understanding of the network, SEanalysis supports interactive visualization of networks using the visualization plugin Echarts (http://echarts.baidu.com).

To view SEs along the genome, we developed a customizable genome browser using JBrowse (http://jbrowse.org) (43) containing more than 6,000 tracks. This browser allows viewing the genomic coordinates of SEs, TF binding sites (TFBS) identified by ChIP-seq, SNPs, DHSs and conservation score. SEanalysis can also link the data to the UCSC genome browser (24).

### Implementation

SEanalysis is freely available to the research community at http://www.licpathway.net/SEanalysis and requires no registration or login. The main framework of SEanalysis was developed based on Java 1.8.0 (https://www.oracle.com/technetwork/java/) and MySQL 5.7.16 (https://www.mysql.com/). JQuery 3.3.1 (http://jquery.com) and Bootstrap 3.3.7 (https://getbootstrap.com/) (an open source front-end framework) were used to design the front-end web interface. Google Chrome, Mozilla Firefox, Opera and Safari are the preferred browsers for display.

## SUMMARY

To provide comprehensive analysis of SE-associated regulatory networks, we designed and developed a novel web server, SEanalysis, with the following functions: (i) Pathway downstream analysis, (ii) Upstream regulatory analysis, (iii) Genomic region annotation. Compared with other SE databases, this webserver focuses on constructing and analyzing the networks between pathways, TFs, SEs, and SE-associated genes. SEanalysis also allows users to readily download SEs for different cells/tissues, in both bed and csv format. The output results of analyses can also be downloaded. In addition, SEanalysis supports external analytical tools of genomic regions such as GREAT (44) and UCSC (24). SEanalysis also links to additional external resources including NCBI Gene (45), GeneCards (46) and UniProt (47).

The rapid development of high-throughput sequencing technology leads to the accelerated accumulation of a large number of epigenomic datasets. SEanalysis will be updated and maintained accordingly. Our effort to establish this web server was prompted by the great need of researchers to understand the biology of epigenomic network regulation. These researchers include cell and molecular biologists, geneticists and data scientists. Moreover, the field of epigenomics is rapidly progressing, and the integrative analysis of epigenomic regulatory networks is one of the most investigated areas. Therefore, SEanalysis will be a valuable resource for experimental and computational biologists in the field of epigenomics.

## Supplementary Material

gkz302_Supplemental_Files
