# Supplementary material for: SEanalysis: a web tool for super-enhancer associated regulatory analysis
Source: Nucleic Acids Res. 2019 Apr 27;47(W1):W248–55. doi: 10.1093/nar/gkz302 (PMC6602466; doi:10.1093/nar/gkz302)
Supplement: gkz302_Supplemental_Files [file gkz302_supplemental_files.zip › Figure S1.pdf]

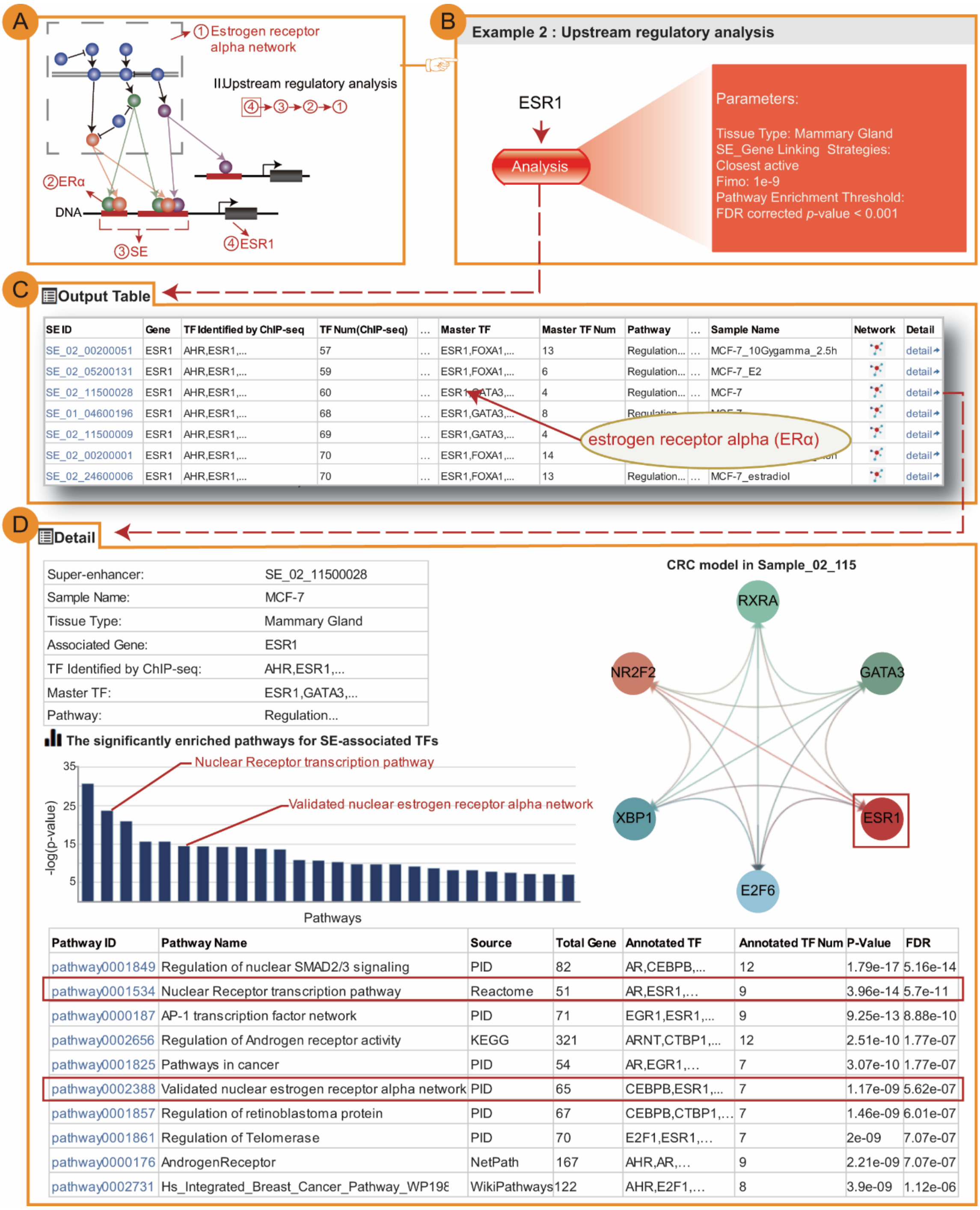

Figure S1. Validation results for 'Upstream regulatory analysis'. (A) Schematic diagram of 'Upstream regulatory analysis'. (B) Input exemplary data and parameters of 'Upstream regulatory analysis'. (C) The output table provided associated SEs, TFs binding to the SEs and upstream pathways. (D) The detailed page of the output table, including the relationships between input genes and identified SEs, TFs binding to the SEs (based on either ChIP-seq data or predicted by motif analysis), master TFs binding to these SEs, and enriched upstream pathways.
